# Recovery Trajectories in Adolescent Girls with Anorexia Nervosa

**DOI:** 10.3390/jcm13030778

**Published:** 2024-01-29

**Authors:** Alexandra Bédard, Catherine Bernard, Dominique Meilleur, Danielle Taddeo, Caroline Pesant, Giuseppina Di Meglio, Nathalie Gingras, Isabelle Thibault, Holly Agostino, Richard Bélanger, Pierre-Olivier Nadeau, Jean-Yves Frappier, Chantal Stheneur, Laurie Dufresne, Catherine Bégin

**Affiliations:** 1 Centre Nutrition, Santé et Société (NUTRISS), Institut sur la Nutrition et les Aliments Fonctionnels (INAF), Université Laval, Québec, QC G1V 0A6, Canada; alexandra.bedard@fsaa.ulaval.ca; 2 École de Psychologie, Université Laval, Québec, QC G1V 0A6, Canada; catherine.bernard.11@ulaval.ca (C.B.); laurie.dufresne.2@ulaval.ca (L.D.); 3 Département de Psychologie, Université de Montréal, Montréal, QC H3C 3J7, Canada; dominique.meilleur@umontreal.ca; 4 Centre Hospitalier Universitaire Sainte-Justine, Montréal, QC H3T 1C5, Canada; danielle.taddeo.med@ssss.gouv.qc.ca (D.T.); pierre.olivier.nadeau.med@ssss.gouv.qc.ca (P.-O.N.); jean-yves.frappier.med@ssss.gouv.qc.ca (J.-Y.F.); chantal.stheneur@umontreal.ca (C.S.); 5 Hôpital Fleurimont, Centre Hospitalier Universitaire de Sherbrooke, Sherbrooke, QC J1H 5H3, Canada; caroline.pesant@usherbrooke.ca; 6 Hôpital de Montréal pour Enfants/Montreal Children’s Hospital, Centre Universitaire de Santé McGill, Montréal, QC H4A 3J1, Canada; giuseppina.dimeglio@mcgill.ca (G.D.M.); holly.agostino.med@ssss.gouv.qc.ca (H.A.); 7 Centre de Pédopsychiatrie, Centre Intégré Universitaire de Santé et de Services Sociaux de la Capitale-Nationale, Québec, QC G1N 2W1, Canada; nathalie.gingras@fmed.ulaval.ca; 8 Département de Psychiatrie et de Neurosciences, Faculté de Médecine, Université Laval, Québec, QC G1V 0A6, Canada; 9 Département de Psychoéducation, Université de Sherbrooke, Sherbrooke, QC J1K 2R1, Canada; isabelle.thibault@usherbrooke.ca; 10 Département de Pédiatrie, Faculté de Médecine, Université Laval, Québec, QC G1V 0A6, Canada; richard.belanger@mail.chudequebec.ca; 11 Centre Hospitalier Universitaire de Québec, Québec, QC G1V 4G2, Canada; 12 Département de Pédiatrie, Faculté de Médecine, Université de Montréal, Montréal, QC H3T 1C5, Canada

**Keywords:** anorexia nervosa, trajectories, predictors, adolescents, body weight, food restriction, excessive exercise, clinical, depressive symptomatology, personality

## Abstract

**Background:** This study aimed to document recovery trajectories among adolescents with anorexia nervosa (AN) based on three markers of remission, namely changes in body weight, food restriction, and excessive exercise, and to identify predictors of these trajectories. **Methods:** One hundred twenty-six adolescent girls (14.7 ± 1.3 years) were recruited during initial assessment visits at specialized eating disorder (ED) programs in five University Health Centers across the province of Quebec, Canada. z-BMI and AN symptom severity (food restriction and excessive exercise) were assessed at initial assessment visits and subsequently reassessed at each quarterly follow-up over a 12-month period to identify recovery trajectories. **Results:** Considering the three markers of remission, three distinct trajectories emerged: Group 1, rapid responders; Group 2, gradual responders; and Group 3, unstable responders. At initial visits, a difference between groups was found regarding the type of treatment (*p* = 0.01) and weight suppression (*p* = 0.02). Group 1 had a higher number of youths hospitalized than Group 2 and Group 3, and a greater weight suppression than Group 3. Furthermore, individuals with atypical AN were more likely to belong to Group 2 than to Group 1 and Group 3 (*p* < 0.0001). **Conclusions:** This study contributes to a better understanding of the heterogeneity of recovery trajectories in adolescent girls with AN.

## 1. Introduction

Anorexia nervosa (AN) is an eating disorder (ED) that typically emerges during adolescence, with a lifetime prevalence of up to 4% among females and 0.3% among males [1,2]. This condition is characterized by cognitive disturbances and maladaptive eating behaviors aimed at controlling body weight [1]. While AN is a fully specified ED, atypical AN is a subtype within the broader category of “other specified feeding and eating disorder”. An atypical AN diagnosis describes individuals who meet all of the criteria for AN, except that despite significant weight loss, the individual’s weight remains within or above the normal range [1]. The serious consequences associated with AN make it a critical public health concern. Malnutrition resulting from AN can lead to significant medical complications such as cardiovascular complications [3], bone mineral density loss [4,5], and impaired linear growth [6,7]. AN is also recognized as having one of the highest mortality rates of any mental health disorders and is associated with several psychiatric comorbidities, such as anxiety disorders and depression [8,9,10]. The prognosis is more favorable when the disease manifests during adolescence and is treated early [11]. As a result, implementing effective interventions during adolescence is of the utmost importance to prevent the long-term consequences of this disorder.

Providing psychological and medical interventions for adolescents with AN is highly challenging due to the significant heterogeneity within this population [12,13]. Previous studies have demonstrated heterogeneity in affective and behavior presentations, as well as in personality traits, during assessments of adolescents with AN [12,14,15,16]. The treatment approaches employed with adolescents suffering from AN, and the patterns of response following treatment also vary significantly [17,18,19]. On that note, a longitudinal study conducted among adolescents revealed that only 41% of the sample experienced complete remission from AN [20].

Recently, some studies have focused on the recovery trajectories during the treatment of AN [17,18,19,21,22,23,24]. Characterizing the recovery trajectories of patients with AN may allow for an improved understanding of the treatment response. Furthermore, identifying predictors of these trajectories would contribute to delivering more personalized care and improving patient outcomes. Among studies that have examined recovery trajectories in individuals with AN, only two considered adolescents, and these studies only focused on body weight as a single marker of remission [17,19]. These two studies clearly illustrated the significant heterogeneity in weight gain trajectories observed in response to treatment. First, Berona et al. [17] identified three distinct trajectories of weight gain among adolescents and young adults with AN or atypical AN during the first five weeks following the beginning of treatment: a slow weight gain trajectory, a moderate weight gain trajectory, and a rapid weight gain trajectory. Lebow et al. [19] also drew trajectories based on weight gain among adolescents with AN or atypical AN. Six months after the beginning of treatment, five distinct weight gain trajectories were found: 1—slow and steady weight gain throughout the intervention; 2—moderate initial weight gain followed by stabilization; 3—rapid weight gain with early stabilization; 4—rapid initial weight gain, followed by a small weight loss, and then weight stabilization; and 5—higher initial BMI-for-age percentile, with no significant weight gain throughout the intervention [19]. Despite the limited number of studies aimed at documenting predictors of weight gain trajectories among adolescents with AN, results from these two previous studies suggest that younger age [19], greater body weight suppression [19], and the absence of a mood disorder diagnosis [17] might be predictors of rapid weight gain trajectories.

In summary, studies documenting adolescent recovery trajectories, although highly informative, have only assessed a short period of remission (≤6 months). Considering that the average remission time to regain a healthy body weight in AN is approximately 9 to 12 months [25], documenting a longer study period of remission could provide more insights to better identify those who still require care or are at risk of relapse. In addition, these studies have been limited to body weight as the sole marker of remission. However, body weight is a result of diverse symptoms present in AN, such as food restriction and excessive exercise, both of which are important treatment targets. These behaviors represent strategies for controlling body weight and coping with negative emotions, and they may contribute to the maintenance of the disorder or potential relapse if not adequately addressed in treatment [26,27,28]. Consequently, incorporating food restriction and excessive exercise, in addition to body weight, into trajectory analyses could offer a more comprehensive understanding of the progression of illness symptoms over time compared to previous weight gain trajectories.

Additionally, besides mood disorders, psychoaffective variables have received limited attention in relation to recovery trajectories among adolescents with AN. Personality traits are particularly important as they measure the characteristics contributing to the distinctive ways individuals think, feel, and behave [29]. More recently, there has been growing interest in the impact of personality traits on AN outcomes. For example, Zerwas et al. [30] found that anxiety and impulsivity traits were associated with a poorer prognosis within a large sample of women with AN. Muzy et al. [31] found that personality disorder features predict treatment success in adults presenting with schizoid, avoidant, and paranoid traits, leading to poorer therapeutic outcomes. However, the impact of personality traits on recovery trajectories among adolescents with AN has not been explored yet. Investigating the relationship between recovery trajectories and personality traits might enable clinicians to tailor AN interventions to individual adolescents.

The objectives of this study were, therefore, to (a) document recovery trajectories over a 12-month period among adolescent girls with AN based on three markers of remission, namely changes in body weight, food restriction, and excessive exercise, and to (b) identify predictors of these trajectories. Age, AN diagnosis (AN/atypical AN), AN subtype (restricting (AN-R) subtype/binge-eating/purging (AN-BP) subtype), duration of illness, prior hospitalization, treatment (inpatient vs. outpatient), body weight suppression, depressive symptomatology, and personality traits were considered as potential predictors.

## 2. Methods

### 2.1. Participants

Between May 2016 and July 2020, adolescents (12–17 years) diagnosed with AN were recruited during initial assessment visits in five hospitals affiliated with specialized ED programs that offer both inpatient and outpatient treatment. These hospitals were located within five University Health Centers across the province of Quebec, Canada. Patients diagnosed with AN or atypical AN by the attending physician (i.e., child and adolescent psychiatrists specialized in ED) according to the DSM-5 criteria [1] were included. Treatment involved interdisciplinary care, encompassing family and individual therapy sessions, nutrition counseling, meal support, psychiatric and medical monitoring, and additional therapeutic interventions (e.g., art therapy).

Before participating in this study, all patients provided their written informed assent, and parents/legal guardians provided written informed consent. This study has been approved by the ethics committee of the coordinating center (CHU de Québec-Université Laval: # MP-20-2015-2323) and by the ethic committees of each participating center.

### 2.2. Measures

#### 2.2.1. Variables Used to Identify Recovery Trajectories

##### Weight-Related Variables

Anthropometric measurements, including body weight (in kg) and height (in m), were measured by the medical team during the initial assessment visits and at 3-month, 6-month, 9-month, and 12-month follow-up appointments. The z-score of BMI (z-BMI) was then calculated for each patient based on the World Health Organization chart [32].

##### Eating Disorder Symptom Severity Scale

The Eating Disorder Symptom Severity Scale (EDS3) [33,34] was completed by the attending physician during the initial assessment visits and at 3-month, 6-month, 9-month, and 12-month follow-up appointments. This instrument assesses the severity of the ED according to five general domains: (1) ED symptoms and behaviors, (2) ED urges, (3) ED cognitions, (4), ED anxiety, and (5) treatment progress. In the present study, two subscales of the ED symptoms and behaviors domain, namely food restriction and excessive exercise, were used. More precisely, the food restriction subscale asks to rate how much and how regularly the youth eats. When answering, the attending physician had to compare the youth’s eating habits to those of same-aged peers with similar activity levels. For the excessive exercise subscale, exercise is considered excessive when it is only about losing weight, when it is to maintain or create a certain body shape, when it occurs on a rigid schedule, and/or when it is no longer about enjoyment. For these two subscales, items were answered on a 4-point scale reflecting the severity level of the specific behavior: absence (0), mild (1), moderate (2), and severe (3). Accordingly, higher scores indicate more severe symptoms. All attending physician participated in a 90 min training session to ensure that the EDS3 was administered accurately and consistently across physicians. The instrument has good psychometric qualities, including adequate test–retest reliability of 0.90 and inter-rater reliability correlation above 0.70 for all trainers [34,35].

#### 2.2.2. Predictors of Recovery Trajectories

##### Demographic and Clinical Characteristics

Demographic (e.g., age) and clinical characteristics (e.g., AN diagnosis (AN/atypical AN), duration of illness, prior hospitalization related to ED, treatment (inpatient or outpatient), body weight suppression ([[body weight at initial assessment visits − past maximal body weight]/past maximal body weight] × 100) were collected during the initial assessment visits by the medical team. AN subtype was also reported by the attending physician during the initial assessment visits. The AN-R subtype referred to patients who severely limit the amount and type of food consumed. On the other hand, the AN-BP subtype referred to individuals who also impose significant restrictions on their food intake but engage in episodes of binge-eating and purging, which involves getting rid of consumed food through actions such as vomiting or using laxatives or diuretics.

##### Depressive Symptomatology

During their initial assessment visits, patients completed the Children’s Depression Inventory-2 (CDI-2) [36]. The CDI-2 is a self-reported questionnaire including 28 items designed to assess symptoms of depression in people aged 7 to 17 years old. Each item includes a group of three statements for which the respondent must select the one that best describes his/her condition in the preceding two weeks, ranging from 0 (no depressive symptom) to 2 (significant depressive symptoms). After reverse-scoring the negatively keyed items, a global severity score was calculated from the sum of the 28 items. Higher scores indicate more severe depressive symptoms. In the present study, we used the T-scores of the global severity scale according to the following guidelines: <40 = low depressive symptoms; 40 to 59 = average; 60 to 64 = high average; 65 to 69 = elevated; ≥70 = very elevated symptoms [36]. For the present study, internal consistency for the global severity scale was 0.92.

##### Personality Traits

During their initial assessment visits, patients also completed the Millon Adolescent Clinical Inventory (MACI) to assess personality traits [37,38]. This self-reported inventory consists of 160 true (0)/false (1) items and includes 31 scales grouped under three categories: (1) personality prototypes, (2) clinical syndromes, and (3) expressed concerns. In this study, only the 12 scales that are related to personality prototypes were used, namely introversive, inhibited, doleful, submissive, dramatizing, egotistic, unruly, forceful, conforming, oppositional, self-demeaning, and borderline tendency. Raw scores were converted to base rate scores, considering age and gender. Base rate scores for personality scales were used in continuous form. Higher scores indicate more problematic personality traits. Scores ranging between 75 and 84 were interpreted as elevated while scores of 85 or higher were interpreted as highly elevated. A validation study on a clinical sample found that internal consistency indexes vary between 0.73 and 0.91, whereas the temporal stability coefficients (test–retest) are adequate, varying between 0.57 and 0.92 [35,38,39]. In the present study, the Cronbach alphas varied between 0.49 and 0.93.

### 2.3. Statistical Analyses

To identify recovery trajectories, group-based multi-trajectory modeling was conducted using SAS (version 9.4, SAS Institute Inc., Cary, NC, USA) with the macro PROC TRAJ [40]. Group-based multi-trajectory modeling is a statistical technique used to identify latent clusters following similar trajectories across multiple outcomes [40]. z-BMI, as well as symptom severity scores related to food restriction and excessive exercise, were determined to have censored normal distributions. PROC TRAJ is designed to handle missing data, requiring a minimum of two observations for each individual [41]. Accordingly, only patients with at least two measures on each variable were included. To identify the best fitting model, a two-stage approach was used [40]. As a first step, two, three, four, and five group models were run to compare the Bayesian information criteria (BIC) of each model, with lower BICs values indicating better model fit [40]. In the second stage, quadratic terms were fitted in the model to determine the shape of each trajectory. Based on Nagin et al. [40], criteria for judging the adequacy of the selected model included the average posterior probability of assignment (APPA > 70%), the odds of correct assignment (OCC > 5), and sufficient group sizes (at least 5% of the sample per group). To identify individual predictors of these recovery trajectories, chi-square tests (for category variables) and analysis of variance (for continuous variables) were subsequently conducted to examine differences between group memberships with respect to age, AN diagnosis, AN subtype, duration of illness, prior hospitalization, treatment (inpatient vs. outpatient), body weight suppression, depressive symptomatology, and personality traits. A *p*-value < 0.05 was considered statistically significant. As the number of participants in each group was small, effect sizes were also calculated using Cohen’s *h* for categorical variables and Cohen’s *d* for continuous variables. An effect size of 0.2 was considered small, 0.5 was considered medium, and 0.8 was considered large [42].

## 3. Results

### 3.1. Demographic and Clinical Characteristics

Among the 215 recruited adolescents, 81 did not have at least two measures for each of the variables included in the recovery trajectory, so they were excluded from this analysis. Given the low number of boys (*n* = 8), they were also excluded from the present analyses. The sample consisted of 126 adolescent girls between 12 and 17 years old (14.7 ± 1.3 years). Most patients were Caucasian (91.4%). Among the included patients, 27.8% were inpatients and 72.2% were outpatients at assessment visits. In addition, 77.0% were diagnosed with typical anorexia and 23.0% with atypical anorexia. Finally, 88.3% were diagnosed with AN-R and 11.7% with AN-BP. No difference was observed between the youth included and excluded from the study, except for the treatment received at the initial assessment visits, individuals included in the present analyses being more likely to be inpatients compared to excluded individuals (29.2% vs. 15.9%; *p* = 0.03).

### 3.2. Trajectories

**Figure 1:**
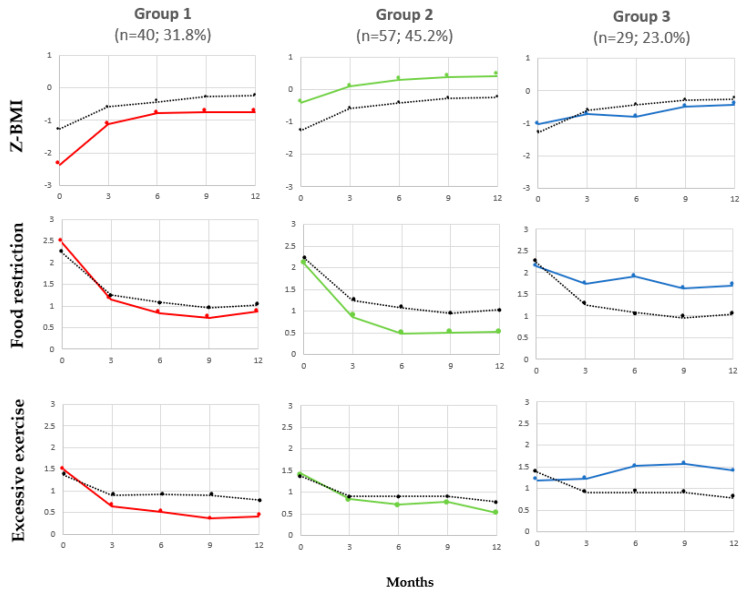
Multi-trajectories of z-BMI, food restriction, and excessive exercise. The dotted lines represent the average for the entire sample. Group 1: rapid responders; Group 2: gradual responders; Group 3: unstable responders. Note. z-BMI labels according to World Health Organization chart: > −2 = Normal, −2 to −3 = Thinness, < −3 = Severe thinness. Food restriction and excessive exercise labels reflecting the severity level of the specific behavior according to the Eating Disorder Symptom Severity Scale (EDS3): 0 = absence, 1 = mild, 2 = moderate, and 3 = severe.

The group-based multi-trajectory modeling analysis resulted in a three-group model (Figure 1). Group 1 (*n* = 40, 31.8%), labeled “rapid responders”, was characterized by a lower initial z-BMI (in the 1st percentile) compared to Group 2 (in the 34th percentile) and Group 3 (in the 15th percentile) and a rapid response to treatment. More precisely, during the first three months, a rapid body weight gain was observed in Group 1, followed by a smaller weight gain between the 3-month and 6-month follow-ups, and then a stabilization period between the 6-month and 12-month follow-ups. The z-BMI for this group consistently remained lower than the average z-BMI of the entire sample at each follow-up measurement (23rd percentile at the 12-month follow-up). Likewise, a marked decrease in food restriction and excessive exercise scores was observed in this group in the first three months, followed by a slight decrease up to the 9-month follow-up. However, a slight increase in both scores was observed at the 12-month follow-up. At the 12-month follow-up, food restriction was still mild (e.g., may sometimes skip a meal and/or refuse certain foods; pay attention to fat and/or caloric content of foods) while excessive exercise was almost null (e.g., do not exercise to control body weight/shape).

Group 2 (*n* = 57, 45.2%), labeled “gradual responders”, was characterized by a higher initial z-BMI (in the 34th percentile) compared to the other two groups. The pattern of z-BMI response was characterized by gradual weight gain and a body weight consistently higher than the average of the entire sample throughout the treatment. At the 12-month follow-up, z-BMI (in the 66th percentile) was the highest compared to the other groups. A marked decrease in food restriction was observed in the first three months, followed by a smaller decrease between the 3-month and 6-month follow-ups, and then a stabilization period between the 6-month and 12-month follow-ups. For excessive exercise, a moderate decrease was observed in the first three months, followed by a slight decrease at the 6-month follow-up, a slight increase at the 9-month follow-up, and subsequently a decrease at the 12-month follow-up. At the 12-month follow-up, the food restriction score was almost null (e.g., eat all meals; do not restrict; may occasionally skip a meal, but make up for it throughout the day; eat a wide variety of food) as was excessive exercise score (e.g., do not exercise to control body weight/shape).

Group 3 (*n* = 29, 23.0%), labeled “unstable responders”, was characterized by a low initial z-BMI (in the 15th percentile; higher than Group 1 but lower than Group 2) and exhibited an unstable pattern of response for z-BMI, food restriction, and excessive exercise. Body weight fluctuated significantly between each follow-up measurement, ending at the 33rd percentile at the 12-month follow-up, which is lower than the average of the entire sample. At the 12-month follow-up, food restriction and excessive exercise scores were the highest compared to the other groups and above the average of the entire sample. Food restriction remained moderate (e.g., frequently skip meals and/or refuse certain food groups, may often hide food, do diets, count calories) and excessive exercise score was between mild and moderate (e.g., exercise sometimes to often to make up for eating or to control body weight/shape).

### 3.3. Predictors of Trajectories

At the initial assessments, there were no significant differences between groups in terms of age, AN subtype (AN-R vs. AN-BP), duration of illness and prior hospitalization. However, a difference was found regarding the AN diagnosis (AN vs. atypical AN; *p* < 0.0001), individuals with atypical AN were more likely to belong to Group 2 “gradual responders” than to Group 1 “rapid responders” and Group 3 “unstable responders”. In addition, a difference between groups was found regarding the type of treatment (inpatient vs. outpatient; *p* = 0.01), Group 1 “rapid responders” having a significantly higher number of youths hospitalized compared to Group 2 “gradual responders” and Group 3 “unstable responders”. A difference between groups was also found regarding body weight suppression (*p* = 0.02), Group 1 “rapid responders” being characterized by a greater weight suppression compared to Group 3 “unstable responders”. Groups did not differ significantly in terms of depressive symptoms and personality traits. However, although not statistically significant, medium effect sizes were found between Group 1 “rapid responders” and Group 3 “unstable responders”, Group 1 being characterized by higher scores for depressive symptoms (Cohen’s *d* = 0.51) and doleful traits (Cohen’s *d* = 0.51) and lower score for egotistic traits (Cohen’s *d* = 0.55) than Group 3. A medium effect size was also noted between Group 2 “gradual responders” and Group 3 “unstable responders” for oppositional traits, Group 2 being characterized by higher scores than Group 3 (Cohen’s *d* = 0.50). Table 1 provides a summary of the comparisons at the initial assessment visits among the three groups.

## 4. Discussion

The present study documented recovery trajectories over a 12-month period among adolescent girls with AN based on three markers of remission, namely body weight gain, decreased food restriction, and the rate of excessive exercise. Considering that the average remission time to regain a healthy body weight in AN is approximately 9 to 12 months [25], assessing the first year of treatment was highly relevant. Moreover, the inclusion of other important treatment targets as markers of remission, in addition to body weight is certainly a strength of the present study, as it provided a more complete picture of patients’ experiences. Our findings suggest three distinct 12-month recovery trajectories: 1—rapid responders; 2—gradual responders; and 3—unstable responders. These recovery trajectories are described below.

Firstly, the «rapid responders» group constituted one third of the sample. During the initial assessment, this group exhibited the lowest z-BMI, the highest body weight suppression, and the most severe food restriction. This group also had a higher proportion of inpatient adolescents and was characterized by elevated depressive symptoms, highlighting a more severe clinical profile compared to the other two groups. The presence of more doleful traits, which highlight a loss of joy and an inclination to pessimism, could be related to the presence of depressive symptoms, but also to the more severe undernutrition in this group. Furthermore, this group exemplifies the relevance of considering not only body weight but also other markers of remission. Although the z-BMI remained low at the 12-month follow-up (in the 23rd percentile), this group was considered to have reached a healthy weight according to WHO charts [32] after three months of treatment, and they maintained this weight gain throughout the follow-up. However, despite decreases in food restriction and excessive exercise during the first nine months, they thereafter experienced an increase between the 9-month and 12-month follow-ups. At the 12-month follow-up, they were still characterized by a mild level of restriction according to their attending physician, suggesting that these adolescent girls may skip meals, refuse certain foods, or pay attention to the fat and caloric content of foods. This observation may illustrate the fragility of this group in maintaining progress over time and suggests the importance of ongoing care beyond achieving a body weight qualified as “healthy” to prevent relapse.

The “gradual responders” group, representing the largest group of adolescents, was characterized by a higher BMI at initial assessment, suggesting a lower level of undernutrition in this group. These adolescent girls experienced also the most favorable progression throughout the follow-up for all three remission markers. At the end of follow-up, the mean z-BMI was in the 66th percentile, and food restriction and excessive exercise scores were almost null, suggesting that this group had the lowest risk of short-term relapse. Interestingly, this group was characterized by a more balanced proportion of adolescents with typical and atypical AN.

Finally, the “unstable responders” group, representing the smallest group of adolescent girls, was characterized by fluctuations in body weight throughout the follow-up. At the end of the 12-month follow-up, their z-BMI remained low (33rd percentile) but was considered “healthy” according to WHO charts [32]. On the other hand, behaviors associated with food restriction and excessive exercise remained present throughout the 12-month follow-up. Once again, these results suggest the importance of looking beyond body weight. Despite achieving a BMI classified as ‘healthy’, these adolescents still appear to require care that specifically addresses AN symptoms to enhance their prognosis. Indeed, the attending physician reported that these adolescent girls continued to frequently skip meals, refuse certain food groups, often hide food, follow diets, or monitor calorie intake at the 12-month follow-up. Additionally, they relied on exercise to compensate for eating or to exert control over body weight and shape.

Taken together, these findings highlight that approximately 45% of adolescent girls, namely those in the “gradual responders” group, exhibit a favorable trajectory during the first 12 months of treatment. These results suggest that adolescent girls with a less severe profile related to markers of remission at the initial assessment visits, and who respond gradually to treatment, may be at lower risk of short-term relapse compared to the other two groups. These findings align with those of Herpertz-Dahlmann et al. [20], where only 41% of adolescents experienced complete remission from AN. However, given the relatively short follow-up in the present study (i.e., 12 months), designing studies that follow adolescents over a longer period of time would be of interest, considering the substantial risk of symptom relapse after the first year of treatment [43,44].

Anorexia subtype (AN-R/AN-BP), duration of illness, and prior hospitalization did not discriminate group membership, which is in line with previous studies on body weight gain trajectory in adolescents with AN [17,19]. Age also did not discriminate group membership, in contrast to the findings of Lebow et al. [19], suggesting that a younger age is associated with more rapid weight gain trajectories. However, as mentioned earlier, there was a difference in the type of treatment (inpatient vs. outpatient) among the three groups, with the “rapid responders” group being more hospitalized at the beginning of treatment than the other two groups. Additionally, the “rapid responders” group was characterized by the highest body weight suppression, replicating the results previously found by Lebow et al. [19], linking greater weight suppression to more rapid weight gain trajectories. Given the scarcity of studies aimed at documenting predictors of recovery trajectories among adolescents with AN, additional research is needed to better understand how the clinical profile of adolescents with AN may influence their recovery trajectory in the short and long term.

These three groups did not significantly differ according to depressive symptoms. However, although not statistically significant, the “rapid responders” group exhibited higher levels of depressive symptoms. These results challenge our initial hypothesis, which posited that the absence of depressive symptoms would be associated with more rapid recovery trajectories. This replicates the result found in adults with AN [45] but differs from a study conducted among adolescents in partial hospitalization, where a depression diagnosis decreased the probability of belonging to a rapid or moderate body weight gain trajectory [17]. The use of a self-reported questionnaire to assess depressive symptoms, rather than an evaluation by a physician as in the study by Berona et al. [17], may explain the difference observed. In addition, these more elevated depressive symptoms in the “rapid responders” group at the initial assessment may be explained by the fact that this group was more clinically deteriorated. This more deteriorated profile may have provided greater room for improvement, potentially explaining why, despite experiencing more severe depressive symptoms, this group responded rapidly to treatment. This is particularly noteworthy, considering that this group was more hospitalized and, as a result, received potentially more intensive care during the first months of therapy. The therapeutic framework in a hospital setting may have played a role in managing depressive symptoms. Further studies will be needed to explore the influence of depressive symptoms on the recovery of adolescents with AN.

The present study was the first to include personality traits as predictors of recovery trajectories among adolescents with AN. The most prominent personality traits were the same for the three groups, namely introverted, inhibited, and submissive traits. However, although not statistically significant, some differences of medium effect sizes were observed between groups for doleful, egotistic, and oppositional traits. These findings suggest the relevance of examining personality traits in further well-powered studies aiming to identify the predictors of recovery trajectories in adolescent girls with AN.

Some limitations need to be considered in the present study. First, the study population was very uniform (white female), and findings may not be extrapolated to other genders and ethnicities. Accordingly, it could be particularly relevant to conduct further studies among boys and adolescents from different ethnic backgrounds. Second, the EDS3 questionnaire that was used to measure food restriction and excessive exercise was a clinician-rated measure, and clinicians may underestimate or overestimate symptom severity. The self-reported nature of some questionnaires could also have introduced recall and desirability bias. Third, even if the present study had a suitable sample size for conducting a group-based multi-trajectory modeling analysis, i.e., a minimum of 100 cases, an optimal range of 300 to 500 cases is preferred [46]. Therefore, the modeling accuracy could have been enhanced with a larger sample size. In addition, our study may have lacked the statistical power to identify predictors, as each group included only 29 to 57 participants. Therefore, other more powered studies would be of relevance to shed some light on the predictors of recovery trajectories among adolescents with AN. Fourth, the inclusion of adolescents with atypical anorexia may have influenced the results. However, since the goal of this study was to obtain a representative picture of the clientele presenting in our services, we believe it was essential to include them in our analyses. Further studies should be conducted comparing adolescents with typical anorexia versus those with atypical anorexia to better understand their respective remission trajectories. Finally, several adolescents were excluded from the analysis due to a high amount of missing data. Missing data in longitudinal studies involving participants with ED is a common issue (e.g., participants leaving the study prematurely (i.e., dropout) or not presenting for medical follow-ups [47]), which was exacerbated in our study by the COVID-19 pandemic (e.g., difficulties in maintaining regular medical follow-ups with participants).

## 5. Conclusions

In summary, this study contributes to a better understanding of the heterogeneity of recovery trajectories in adolescent girls with AN, revealing distinct groups. Considering the three markers of remission, namely changes in body weight, food restriction, and excessive exercise, the present study identified three recovery trajectories over 12 months: rapid responders, gradual responders, and unstable responders. Findings indicate that adolescent girls exhibiting a less severe profile in relation to remission markers at the initial assessment visits, and who demonstrate a gradual response to treatment (referred to as the “gradual responders” group), may face a reduced risk of short-term relapse compared to the other two groups. Although some clinical and psychological variables have been related to recovery in the present study, such as AN diagnosis, type of treatment (inpatient vs. outpatient), body weight suppression, depressive symptoms, and personality traits, it would be relevant in future research to consider other variables to distinguish trajectories and, subsequently, to adapt treatment accordingly. For example, family-related variables are an interesting avenue, as the literature shows that the relationship with parents can influence intervention outcomes [48].

## Figures and Tables

**Table 1:** Proportions and mean scores for potential predictors in the three distinct multi-trajectory groups: bivariate analysis.

	Group 1 Rapid Responders (*n* = 40; 31.8%)	Group 2 Gradual Responders (*n* = 57; 45.2%)	Group 3 Unstable Responders (*n* = 29; 23.0%)	*p*-Value	Effect Size
Age (months)	183.58 (18.30)	179.44 (15.53)	185.97 (11.55)	0.16	Groups 1 vs. 2: 0.24
					Groups 1 vs. 3: 0.16
					Groups 2 vs. 3: 0.48
AN diagnosis				<0.0001	Groups 1 vs. 2: 1.41 *
Typical AN	100%	57.9%	82.8%		Groups 1 vs. 3: 0.86 *
Atypical AN	0%	42.1%	17.2%		Groups 2 vs. 3: 0.56 *
AN subtype				0.92	Groups 1 vs. 2: 0.08
AN-R	89.7%	87.0%	88.9%		Groups 1 vs. 3: 0.03
AN-BP	10.3%	13.0%	11.1%		Groups 2 vs. 3: 0.06
Duration of illness (days)	460 (425)	376 (416)	385 (374)	0.67	Groups 1 vs. 2: 0.20
					Groups 1 vs. 3: 0.19
					Groups 2 vs. 3: 0.02
Prior hospitalization				0.44	
Yes	18.9%	9.1%	23.1%		Groups 1 vs. 2: 0.29
No	73.0%	79.5%	61.5%		Groups 1 vs. 3: 0.10
N/D	8.1%	11.4%	15.4%		Groups 2 vs. 3: 0.39
Treatment at initial assessment				0.01	Groups 1 vs. 2: 0.76 *
Inpatient	55.0%	19.3%	20.7%		Groups 1 vs. 3: 0.73 *
Outpatient	45.0%	80.7%	79.3%		Groups 2 vs. 3: 0.04
Weight suppression (%)	−19.8 (7.3)	−15.3 (12.1)	−13.1 (9.1)	0.02	Groups 1 vs. 2: 0.45
					Groups 1 vs. 3: 0.81 *
					Groups 2 vs. 3: 0.21
Depressive symptoms	69.15 (13.08)	65.34 (15.44)	62.30 (13.60)	0.15	Groups 1 vs. 2: 0.27
					Groups 1 vs. 3: 0.51 *
					Groups 2 vs. 3: 0.21
Personality traits					
Introversive	76.48 (20.69)	70.41 (18.46)	73.25 (17.31)	0.32	Groups 1 vs. 2: 0.31
					Groups 1 vs. 3: 0.17
					Groups 2 vs. 3: 0.16
Inhibited	73.51 (14.32)	70.81 (15.63)	72.03 (15.69)	0.70	Groups 1 vs. 2: 0.18
					Groups 1 vs. 3: 0.10
					Groups 2 vs. 3: 0.08
Doleful	66.92 (24.57)	61.11 (30.13)	53.29 (28.89)	0.14	Groups 1 vs. 2: 0.21
					Groups 1 vs. 3: 0.51 *
					Groups 2 vs. 3: 0.26
Submissive	74.44 (14.26)	73.94 (12.39)	74.50 (17.38)	0.98	Groups 1 vs. 2: 0.04
					Groups 1 vs. 3: 0.00
					Groups 2 vs. 3: 0.04
Dramatizing	37.05 (20.46)	44.71 (25.69)	44.14 (25.18)	0.28	Groups 1 vs. 2: 0.33
					Groups 1 vs. 3: 0.31
					Groups 2 vs. 3: 0.02
Egotistic	23.05 (14.09)	30.74 (19.90)	32.79 (20.85)	0.06	Groups 1 vs. 2: 0.45
					Groups 1 vs. 3: 0.55 *
					Groups 2 vs. 3: 0.10
Unruly	27.90 (16.84)	34.53 (20.75)	32.36 (19.30)	0.26	Groups 1 vs. 2: 0.35
					Groups 1 vs. 3: 0.25
					Groups 2 vs. 3: 0.11
Forceful	13.29 (14.93)	18.60 (16.80)	14.07 (16.05)	0.24	Groups 1 vs. 2: 0.33
					Groups 1 vs. 3: 0.05
					Groups 2 vs. 3: 0.28
Conforming	52.85 (12.69)	57.06 (15.41)	57.75 (15.97)	0.30	Groups 1 vs. 2: 0.30
					Groups 1 vs. 3: 0.34
					Groups 2 vs. 3: 0.04
Oppositional	48.00 (22.09)	50.72 (21.19)	39.14 (24.72)	0.09	Groups 1 vs. 2: 0.13
					Groups 1 vs. 3: 0.38
					Groups 2 vs. 3: 0.50 *
Self-demeaning	67.10 (16.12)	62.91 (18.28)	58.21 (22.11)	0.16	Groups 1 vs. 2: 0.24
					Groups 1 vs. 3: 0.46
					Groups 2 vs. 3: 0.23
Borderline tendency	48.74 (22.31)	45.83 (19.73)	39.64 (19.41)	0.20	Groups 1 vs. 2: 0.14
					Groups 1 vs. 3: 0.44
					Groups 2 vs. 3: 0.32

Percentages displayed in chi-square analysis; means and (standard deviations) presented in analysis of variance, with *p* < 0.05. Effect sizes were calculated using Cohen’s *h* for categorical variables and Cohen’s *d* for continuous variables. An effect size of 0.2 was considered small, 0.5 medium, and 0.8 large. * Medium or large effect sizes.

## Data Availability

The data that support the findings of this study are available on request from the corresponding author. The data are not publicly available due to privacy or ethical restrictions.
